# Plasma Concentration of Tumor Necrosis Factor-Stimulated Gene-6 as a Novel Diagnostic and 3-Month Prognostic Indicator in Non-Cardioembolic Acute Ischemic Stroke

**DOI:** 10.3389/fimmu.2022.713379

**Published:** 2022-02-10

**Authors:** Yewei Qu, Fan Yang, Fanwei Meng, Xi Chen, Qingqing Zhang, Tian Yu, Shirong Wen, Yujun Pan

**Affiliations:** Department of Neurology, the First Affiliated Hospital of Harbin Medical University, Harbin, China

**Keywords:** prognosis, protein–protein interaction network, neutrophil, acute ischemic stroke, interleukin-8, tumor necrosis factor-stimulated gene-6

## Abstract

**Background:**

Tumor necrosis factor-stimulated gene-6 (TSG-6) is a multifunctional, anti-inflammatory, and protective protein, while the association between TSG-6 and acute ischemic stroke (AIS) remains unclear in humans. This study aims to investigate the potential diagnostic and short-term prognosis predictive values of TSG-6 in non-cardioembolic AIS.

**Methods:**

A total of 134 non-cardioembolic AIS patients within 24 h after AIS onset and 40 control subjects were recruited. Using an AIS dataset from the Gene Expression Omnibus database and setting the median expression level of *TNFAIP6* as the cutoff point, data were divided into *TNFAIP6*-high and *TNFAIP6*-low expression groups. Differently expressed genes (DEGs) were extracted to perform gene enrichment analysis and protein–protein interaction (PPI) network. Baseline data were analyzed in a four-group comparison plotted as plasma TSG-6 concentration median and 25th/75th percentiles. The correlative factors of 3-month outcome were evaluated by logistic regression. TSG-6 concentrations and TSG-6-to-interleukin-8 ratios were compared in a block design. A receiver-operating characteristic curve was used to analyze the detective value of TSG-6 and 3-month prognosis predictive values of TSG-6 and TSG-6-to-interleukin-8 ratio.

**Results:**

Non-cardioembolic AIS patients had significantly higher plasma TSG-6 levels than control subjects (*P* < 0.0001). The large-artery atherosclerosis group had significantly higher TSG-6 levels than the small-artery occlusion group (*P* = 0.0184). Seven hundred and eighty-two DEGs might be both AIS-related and *TNFAIP6*-correlated genes, and 17 targets were deemed AIS-related being closely relevant to TNFAIP6. Interleukin-8 was selected for further study. The National Institutes of Health Stroke Scale and the Acute Stroke Registry and Analysis of Lausanne scores at admission, lesion volume, neutrophil count, neutrophil-to-lymphocyte ratio, and interleukin-8 level were positively correlated with TSG-6 level, respectively (*P* < 0.0001). The unfavorable outcome group had meaningfully higher TSG-6 levels (*P* < 0.0001) and lower TSG-6-to-interleukin-8 ratios (*P* < 0.0001) than the favorable outcome group. After adjusting for confounding variables, elevated TSG-6 levels remained independently associated with 3-month poor prognosis of non-cardioembolic AIS (*P* = 0.017). In non-cardioembolic AIS, the cutoff values of TSG-6 concentration for detection and 3-month prognosis prediction and the TSG-6-to-interleukin-8 ratio for the 3-month prognosis prediction were 8.13 ng/ml [AUC, 0.774 (0.686–0.861); *P* < 0.0001], 10.21 ng/ml [AUC, 0.795 (0.702–0.887); *P* < 0.0001], and 1.505 [AUC, 0.873 (0.795–0.951); *P* < 0.0001].

**Conclusions:**

Plasma TSG-6 concentration was a novel indicator for non-cardioembolic AIS diagnosis and 3-month prognosis. Elevated TSG-6-to-interleukin-8 ratio might suggest a 3-month favorable outcome.

## Background

Stroke remains to be a major leading cause of global disease burden (1). From 1990 to 2019, the globally absolute number of incident strokes increased by 70%, prevalent strokes increased by 85%, deaths from stroke increased by 43%, and disability-adjusted life-years due to stroke increased by 32% (1). In China, large amounts of medical and health resources have been devoted to patients with stroke (2). Meanwhile, around 795,000 people experience a first-ever or recurrent stroke every year in the USA (3).

Acute ischemic stroke (AIS) triggers an inflammatory cascade accompanied with a series of functional modulations in inflammatory cells (4, 5). Tumor necrosis factor-stimulated gene-6 (TSG-6) encoded by the *TNFAIP6* gene is a multifunctional secretory protein exhibiting anti-inflammatory and tissue protective properties and being implicated in certain physiological processes and some disease pathology (6). Clinical studies have indicated that plasma TSG-6 levels evidently increased in patients with acute coronary syndrome (ACS) (7) and abdominal aortic aneurysms (AAA) (8). In addition, animal studies, as well as humans studies, showed increasing expression of TSG-6 in infarcted brain tissues (9, 10).

Given these findings above, plasma TSG-6 levels may be associated with the occurrence and prognosis of AIS. However, to date, there is little information on the association between circulating TSG-6 and AIS in human. To better understand an emerging role of TSG-6 in non-cardioembolic AIS, we prospectively investigated their association, including the values on the diagnosis and prognosis.

## Methods

**Figure 1:**
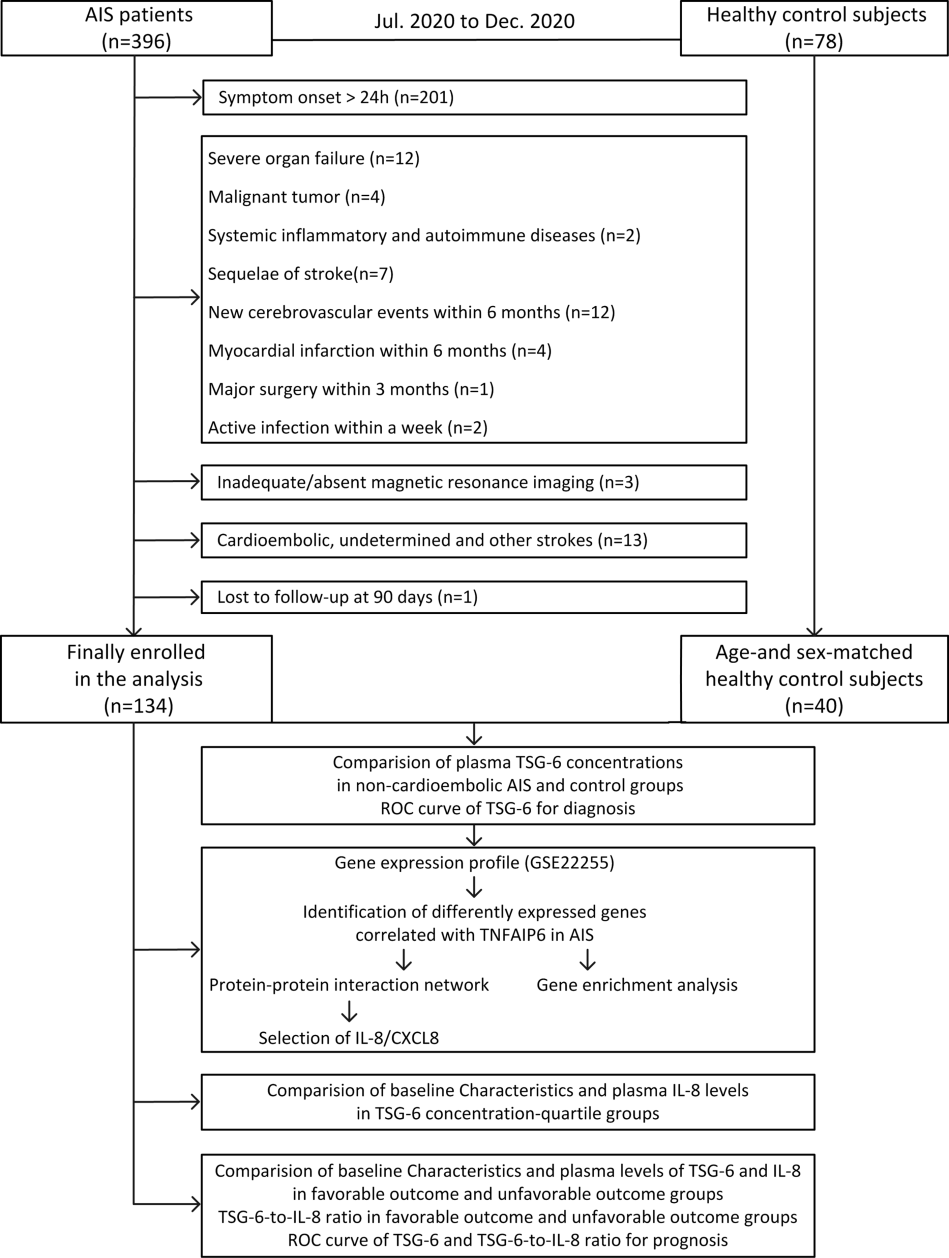
Study flowchart. AIS, acute ischemic stroke; TSG-6, tumor necrosis factor-stimulated gene-6; Roc,receiver-operating characteristic curve; IL-8, interleukin-8.

The flowchart in this study is shown in **Figure 1**.

### Study Population and Evaluation of Clinical Data

A cross-sectional study of the association between plasma TSG-6 levels and non-cardioembolic AIS was conducted. A longitudinal descriptive study was conducted among the non-cardioembolic AIS. The recruitment period was 6 months, from July to December in 2020, in the neurology department at the First Affiliated Hospital of Harbin Medical University. Our study was approved by the local Ethics Committee of the First Affiliated Hospital of Harbin Medical University. AIS was diagnosed according to the World Health Organization criteria (11). AIS patients within 24 hours from symptoms onset were enrolled in the analysis. The exclusion criteria included symptom over 24h, new cerebrovascular events or ACS within 6 months, sequelae of stroke, major trauma or surgery within 3 months, severe organ failure, vascular malformation, malignant tumors, systemic inflammatory and autoimmune diseases, active infection/take antibiotics or antivirals within a week, cardioembolic/undetermined/other strokes, inadequate/absent MRI and lost to follow-up at 90 days. During the same period, people who aimed to take the physical examination in our ward, were collected as healthy control subjects. The exclusion criteria of healthy control group were the same as that of non-cardioembolic AIS group. Stata v15.1 (Stata Corp, College Station, TX) (12) was used to match sex and age between control subjects and non-cardioembolic AIS patients. All participants or their relatives in our study were informed of the study and signed the informed consents.

The clinical characteristics of the patients included sex, age, body mass index (BMI), history of risk factors [including a previous transient ischemic attack (TIA) or symptomatic stroke, hypertension, diabetes mellitus, cardiovascular disease (CVD), alcohol drinking, current smoker], and blood pressure [including systolic blood pressure (SBP), diastolic blood pressure (DBP)]. Neurological deficit at admission was assessed using the National Institutes of Health Stroke Scale (NIHSS) (13) and the Acute Stroke Registry and Analysis of Lausanne (ASTRAL) score (14) to reflect the severity of AIS. According to the Trial of Org 10172 in the Acute Stroke Treatment (TOAST) system (15), non-cardioembolic AIS is divided into large-artery atherosclerosis (LAA) or small-vessel occlusion (SVO). The lesion volume was calculated using the formula 0.5 × *a* × *b* × *c* (16). In detail, “*a*” represents the maximal longitudinal largest diameter, “*b*” represents a second maximal diameter perpendicular to *a*, and “*c*” represents the height of the ellipsoid (5-mm slices containing the infarct were used in the present study). All blood examinations were determined at the Department of Laboratory Medicine of our hospital blinded to baseline data. Acute therapies included mono-antiplatelet therapy, dual antiplatelet therapy (DAPT), statin therapy, intravenous thrombolysis, and endovascular therapy (mechanical thrombectomy, angioplasty, and stenting). Modified thrombolysis in cerebral infarction (mTICI) score was used to assess recanalization after ET and successful reperfusion signified filling of 50% or more of the downstream territory (≥mTICI2b) (17).

### Determination of Circulating TSG-6 and Interleukin-8 (IL-8/CXCL8) Concentration

The cubital venous blood of 3 ml in the AIS group was collected on admission within 24 h from stroke onset, and fasting venous blood was collected into EDTA tubes in the control group. All blood samples were collected into EDTA tubes. After standing for 30 min and centrifugation at 1,500 rpm for 20 min at 4°C, the plasma of the samples was stored at −80°C before the assay immediately. Double antibody sandwich enzyme-linked immunosorbent assay (ELISA) was performed following the instructions of human TSG-6 ELISA kit (RayBiotech, USA) and human IL-8 ELISA kit (ABclonal, China). The results were measured with EMax Plus microplate reader by Molecular Devices. Standard curves (**Supplementary Figure 1**) were drawn for calculating the concentrations of TSG-6 and IL-8. Experienced laboratory technicians who measured TSG-6 and IL-8 concentrations were blinded to the clinical characteristics and outcomes of the participants.

### Data Processing and Identification of Differentially Expressed Genes

The normalized GSE22255 expression data were downloaded from the Gene Expression Omnibus (GEO) database (http://www.ncbi.nlm.nih.gov/geo/) (18). The series of GSE22255 is based on the GPL570 platform (Affymetrix Human Genome U133 Plus 2.0 Array). We extracted the expression data of 20 AIS samples from the GSE22255 dataset. After annotating gene symbols of probes on the GPL570 platform and filtering out the probes without corresponding gene symbols or with multiple genes, we averaged the expression value of genes that have multiple probes mapped. Differential expression analysis was performed using a limma package in R (19). The AIS patients were divided into *TNFAIP6*-high and *TNFAIP6*-low expression groups after setting the median expression level of *TNFAIP6* as the cutoff point. The differentially expressed genes (DEGs) between *TNFAIP6*-high and *TNFAIP6*-low were identified using lmFit and eBayes functions in limma package. The cutoff value was set at *P <*0.05 adjusted by the false discovery rate (FDR) and the absolute value of log_2_-fold change (|log_2_ FC|) ≥0.5.

### Gene Enrichment Analysis and Protein–Protein Interaction Network Construction

The Metascape online tool (http://metascape.org/gp/index.html#/main/step1) (20) was applied to perform the Kyoto Encyclopedia of Genes and Genomes (KEGG) pathway and Gene Ontology (GO) function (biological process, cellular component, and molecular function) analysis for the enrichment analysis of the DEGs. The cutoff value was set at *P <*0.05. The corresponding protein–protein interaction (PPI) network of the DEGs was constructed by STRING (https://string-db.org/) (21). Subsequently, the network was visualized and further processed to assess the core targets interacted with TSG-6/TNFAIP6 by Cytoscape software (v3.6.1, http://www.cytoscape.org/) (22). A node with a high degree of closeness values indicates that the node plays a crucial role in the network. IL-8 was selected for further study.

### Follow-Up and End Point

A 3-month follow-up was performed using the modified Rankin Scale (mRS) (23) by a trained medical staff blinded to clinical and neuroimaging data. At the time of discharge, mRS score from 0 to 2 was defined as a favorable outcome, whereas more than 2 points was adverse.

### Statistical Analysis

The results are expressed as percentages for categorical variables, the means ± standard deviation (SD) for normal continuous variables, and medians [interquartile ranges (IQRs)] for non-normal continuous variables. Continuous data were checked for normal distribution using the Kolmogorov–Smirnov test and Shapiro–Wilk test. Homogeneity of variance used Levene’s test. Proportions were compared using the chi-squared test. Normally distributed continuous variables with equal variance in the two datasets were analyzed by Student’s *t*-test; if not, the Mann–Whitney *U* test was used. A four-group comparison was plotted as plasma TSG-6 concentration median and 25th/75th percentiles. The normally distributed continuous variables with equal variance used the Student–Newman–Keuls *q* test, and the Kruskal–Wallis test was used for statistical evaluation of the rest variables. For bivariate correlations, Spearman’s rank correlation was used. Comparison between the different outcomes was investigated with logistic regression models. We used crude models and multivariable models adjusted for all predictors significant on univariate analysis, and the results were reported as odds ratios (ORs). The receiver-operating characteristic (ROC) curve was used to analyze the predictive value of plasma TSG-6 concentration and 3-month prognosis predictive values of plasma TSG-6 concentration and TSG-6-to-interleukin-8 ratio in non-cardioembolic AIS, and the results were reported as area under the curve (AUC). Two-tailed *P <*0.05 was considered statistically significant. All statistical analyses and graphs were conducted by Statistical Package for Social Sciences (SPSS) version 22 (IBM Corp., Armonk, NY, USA) and GraphPad Prism software version 6.

## Results

### Baseline Characteristics and Plasma TSG-6 Levels in Non-Cardioembolic AIS

In this period, a total of 396 AIS patients were confirmed. Finally, after screening a consecutive range of AIS patients by inclusion and exclusion criteria and follow-up completion, 262 patients were excluded (**Figure 1**) and 134 non-cardioembolic AIS patients were enrolled. During the same period, 78 were collected as healthy control subjects, and of these patients, 40 age- and sex-matched people were included in our study.

**Figure 2:**
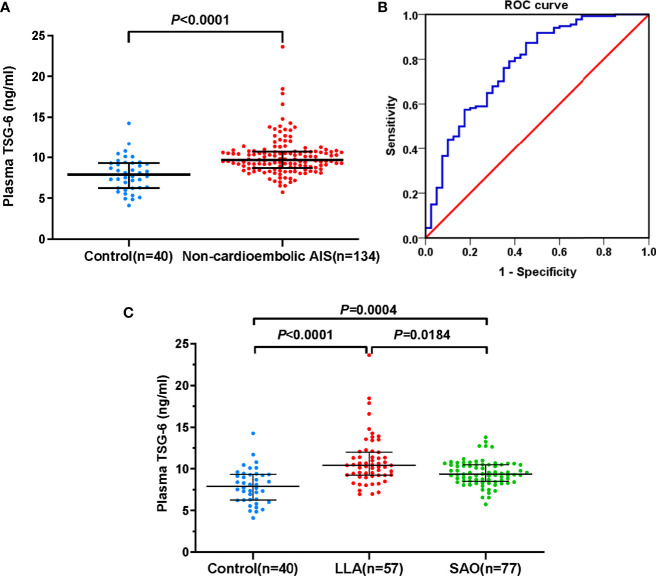
Plasma TSG-6 levels. **(A)** Control subjects and non-cardioembolic AIS. **(B)** The ROC curve of plasma TSG-6 concentration for detecting non-cardioembolic AIS. The AUC value is 0.774 (95% CI, 0.686–0.861; *P* < 0.0001). **(C)** Control subjects, LLA, and SAO AIS. Horizontal lines represent median levels and interquartile ranges. *P*-values determined by the Mann–Whitney *U* test **(A)** and Kruskal–Wallis test **(C)**. AIS, acute ischemic stroke; TSG-6, tumor necrosis factor-stimulated gene-6; LLA, large-artery atherosclerosis; SAO, small-artery occlusion; ROC, receiver-operating characteristic; AUC, area under the curve.

The baseline characteristics of non-cardioembolic AIS patients are presented in **Supplementary Table 1**. At admission, the median TSG-6 concentration in non-cardioembolic AIS group was 9.65 (IQR, 8.71–10.73) ng/ml and significantly higher than that of the controls [7.90 (IQR, 6.26–9.31) ng/ml; *P* < 0.0001; **Figure 2A**], suggesting that high TSG-6 level was associated with non-cardioembolic AIS. The cutoff value for detecting non-cardioembolic AIS (8.13 ng/ml) by the ROC curve gained a relatively high true-positive rate (sensitivity, 0.873) with a moderate false-positive rate (1-specificity, 0.450). The AUC value was 0.774 (95% CI, 0.686–0.861; *P* < 0.0001; **Figure 2B**). According to the TOAST classification, 57 and 77 exhibited LAA and SAO. In the LLA group, there were 36 (63.16%) with only intracranial stenosis, 3 (5.26%) with only extracranial stenosis, and 18 (31.58%) with both intracranial and extracranial stenosis. Comparing the LLA [10.78 (IQR, 10.42–12.25) ng/ml; *P* < 0.0001; **Figure 2C**) or SAO [9.35 (IQR, 8.49–10.50) ng/ml; *P* = 0.0004; **Figure 2C**] groups with the control group, the plasma TSG-6 levels of both subtypes were significantly higher. Additionally, the TSG-6 levels in LLA patients significantly increased than those in SAO patients (*P* = 0.0184; **Figure 2C**).

### Identification of DEGs Correlated With TNFAIP6 in AIS, Gene Enrichment Analysis, and Integration of the PPI Network

**Figure 3:**
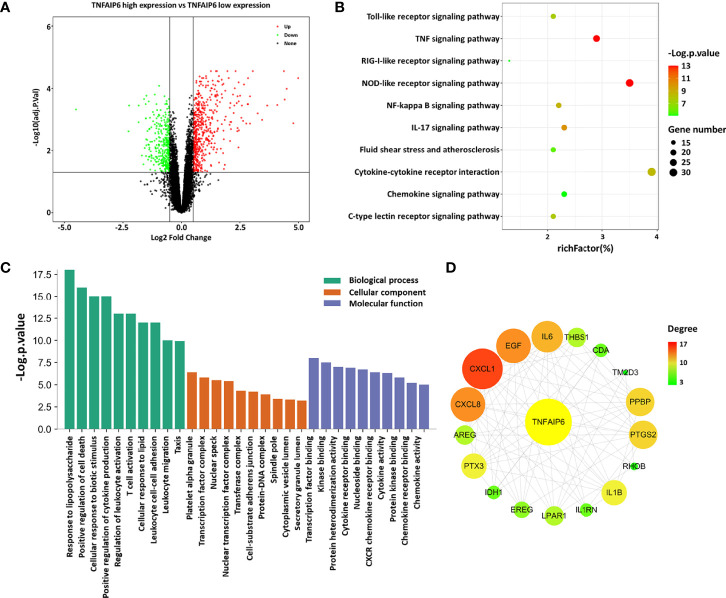
Analysis of differently expressed genes. **(A)** Volcano plot of *TNFAIP6* low/high: green represents downregulated genes, red represents upregulated genes, and black represents no significant differently expressed genes. **(B)** KEGG pathways analysis: the enriched significance gradually increases from green to red, and the dot sizes indicate the number of differential genes contained in the corresponding pathway. **(C)** Enriched GO terms. **(D)** Protein–protein interaction network: circles represent gene-encoded proteins, the related degree gradually increases from green to red, and the circle sizes indicate the target-related degree to TNFAIP. AIS, acute ischemic stroke; KEGG, Kyoto Encyclopedia of Genes and Genomes; GO, Gene Ontology; PPI, protein–protein interaction.

The normalized GSE22255 expression matrix containing 54,675 probes was determined after annotation based on the GPL570 platform. After setting the cutoff value as *P <*0.05 adjusted by the FDR and |log_2_ FC| ≥0.5, a total of 782 DEGs, consisting of 466 upregulated and 316 downregulated genes, were screened out, which may be AIS-related genes that correlate with *TNFAIP6* expression (**Figure 3A** and **Supplementary Data Sheet 1**).

Subsequently, KEGG analysis indicated that the DEGs were involved in pathways associated with response to immunoinflammatory response (NOD-like receptor signaling pathway, IL-17 signaling pathway, c-type lectin receptor signaling pathway, Toll-like receptor signaling pathway, RIG-I-like receptor signaling pathway, chemokine signaling pathway), environmental information processing (TNF signaling pathway, NF-kappa B signaling pathway, cytokine–cytokine receptor interaction), and fluid shear stress and atherosclerosis (**Figure 3B**). Besides, the related biological processes consist of response to biotic stimulus and lipid, leukocyte functional activity, and regulation of cell death and cytokine production. The molecular functions were found mainly in transcription, kinase binding, protein heterodimerization activity, and activity or receptor binding of cytokine and chemokine (**Figure 3C**).

After constructing the PPI network of the DEGs with a threshold of medium confidence not less than 0.4 by STRING and then processing by the Cytoscape software, 17 targets at first-stage nodes were deemed as closely interacting with TSG-6/TNFAIP6 in AIS. We used the cytoHubba plugin to calculate the degree of these targets and the top 3 were CXCL1, CXCL8/IL-8, and EGF (**Figure 3D** and **Supplementary Table 2**).

### Baseline Characteristics and Clinical Variables in Relation to TSG-6 Level

Baseline characteristics of non-cardioembolic AIS patients were stratified in comparison to TSG-6 quartiles in **Supplementary Table 1**. The median concentration of TSG-6 was 9.65 (IQR, 8.71–10.73) ng/ml. Twenty-five percent of the patients had a TSG-6 concentration >10.73 ng/ml, defining the upper quartile. Compared with patients with lower TSG-6 levels, those with higher TSG-6 levels had significant higher NIHSS and ASTRAL scores at admission, lesion volumes, neutrophil counts, neutrophil-to-lymphocyte ratios (NLRs), levels of HCY and IL-8, and lower lymphocyte counts. Plasma TSG-6 level was moderately positively correlated with NIHSS score at admission (*r* = 0.493, *P* < 0.0001) and ASTRAL score at admission (*r* = 0.434, *P* < 0.0001), lesion volume (*r* = 0.359, *P* < 0.0001), neutrophil count (*r* = 0.357, *P* < 0.0001), and NLR (*r* = 0.368, *P* < 0.0001). Moreover, there was a more significant positive correlation between the levels of TSG-6 level and those of IL-8 level (*r* = 0.639, *P* < 0.0001). There was a weak negative correlation between the levels of TSG-6 and those of lymphocyte count (*r* = −0.226, *P* = 0.009). Statistical analysis revealed no significant correlations between TSG-6 level and those of HDL-c and HCY levels (**Table 1**).

**Table 1:** Analysis of relative factors of TSG-6 plasma level in non-cardioembolic AIS patients.

Relevant Factors	*r*_s_	*P*-value
NIHSS at admission	0.493	<0.0001*
ASTRAL at admission	0.434	<0.0001*
Lesion volumes	0.359	<0.0001*
Neutrophil	0.357	<0.0001*
Lymphocyte	-0.226	0.009*
Neutrophil-to-lymphocyte ratio	0.368	<0.0001*
HDL-c	-0.002	0.984
Homocysteine	0.134	0.123
Interleukin-8	0.639	<0.0001*

*P < 0.05.

AIS, acute ischemic stroke; TSG-6, tumor necrosis factor-stimulated gene-6; NIHSS, National Institutes of Health Stroke Scale; ASTRAL, Acute Stroke Registry and Analysis of Lausanne; HDL-c, high-density lipoprotein-cholesterol.

### Associations Between Plasma TSG-6 Level and Non-Cardioembolic AIS Prognosis

**Figure 4:**
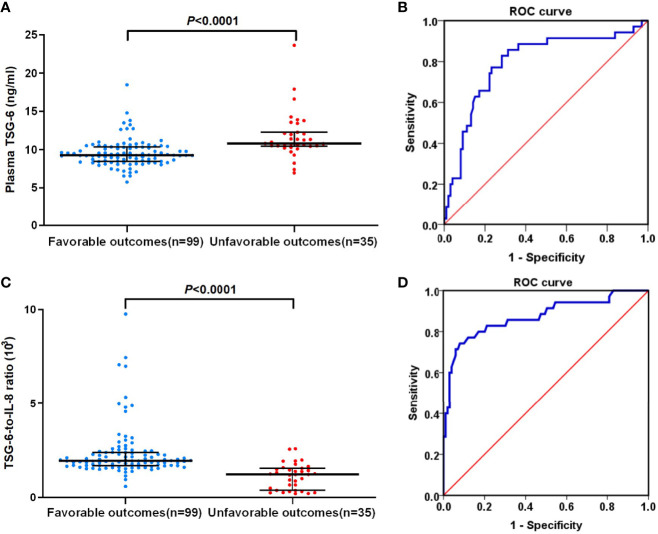
**(A)** Plasma TSG-6 levels between favorable outcome and unfavorable outcome groups in non-cardioembolic AIS. **(B)** The ROC curve of plasma TSG-6 concentration for predicting 3-month prognosis of non-cardioembolic AIS. The AUC value is 0.795 (95% CI, 0.702–0.887; *P* < 0.0001). **(C)** TSG-6-to-IL-8 ratios between favorable outcome and unfavorable outcome groups in non-cardioembolic AIS. **(D)** The ROC curve of TSG-6-to-IL-8 ratio for predicting 3-month-prognosis of non-cardioembolic AIS. The AUC value is 0.873 (95% CI, 0.795–0.951; *P* < 0.0001). The horizontal lines indicate median levels and interquartile ranges. *P*-values determined by the Mann–Whitney *U* test. AIS, acute ischemic stroke; TSG-6, tumor necrosis factor-stimulated gene-6; IL-8, interleukin-8; ROC, receiver-operator characteristic.

An unfavorable outcome was found in 35 (26.12%) patients with an mRS score over 2, and 99 patients got favorable outcomes. In univariate logistic regression analysis, we calculated the ORs of TSG-6 (OR = 1.157; 95% CI, 1.249–1.975; *P* < 0.0001) and other risk factors as presented in **Table 2**. After adjusting for confounding variables (age, alcohol drinking, NIHSS and ASTRAL score at admission, lesion volume, neutrophil and lymphocyte counts, NLR, D-dimer, triglycerides, apolipoprotein A, IL-8, DAPT, and ET), TSG-6 concentration remained an independent predictor of 3-month poor prognosis (OR = 2.171; 95% CI, 1.152–4.093; *P* = 0.017) by multivariable logistic analysis. In addition, lesion volume, neutrophil and lymphocyte counts, NLR, and IL-8 level remained independently significant outcome predictors (**Table 2**). In patients with unfavorable functional outcomes, plasma TSG-6 levels were meaningfully higher compared with those in patients with favorable outcomes [10.78 (IQR, 10.42–12.25) vs. 9.28 (IQR, 8.44–10.34) ng/ml; *P* < 0.0001; **Figure 4A**).

**Table 2:** Logistic regression analysis for the 3-month outcome.

Parameter	Univariate Analysis		Multivariable Analysis	
	OR (95% CI)	*P-*value	OR (95% CI)	*P-*value
Sex	1.544 (0.672–3.550)	0.304		
Age	1.051 (1.008–1.095)	0.019	1.150 (0.956–1.382)	0.138
BMI	0.987 (0.879–1.108)	0.825		
Previous stroke/TIA	1.250 (0.571–2.736)	0.577		
Hypertension	1.095 (0.488–2.460)	0.826		
Diabetes mellitus	1.576 (0.697–3.563)	0.275		
Cardiovascular disease	1.248 (0.490–3.175)	0.643		
Alcohol drinking	0.418 (0.189–0.924)	0.031	0.975 (0.048–19.774)	0.987
Current smoker	0.912 (0.420–1.979)	0.815		
SBP	0.996 (0.981–1.012)	0.635		
DBP	0.976 (0.950–1.004)	0.092		
NIHSS at admission	1.275 (1.167–1.393)	<0.0001*	1.011 (0.416–2.457)	0.980
ASTRAL at admission	1.229 (1.143–1.322)	<0.0001*	1.12 (0.488–2.606)	0.779
Lesion volume	1.017 (1.009–1.025)	<0.0001*	1.042 (1.013–1.073)	0.005*
Neutrophil	1.485 (1.239–1.781)	<0.0001*	8.644 (1.482–50.422)	0.017*
Lymphocyte, 10^9^/L	0.160 (0.061–0.420)	<0.0001*	0.000 (0.000–0.043)	0.013*
Neutrophil-to-lymphocyte ratio	1.253 (1.106–1.419)	<0.0001*	0.155 (0.030–0.798)	0.026*
Platelet	0.993 (0.986–1.000)	0.061		
Fibrinogen	1.320 (0.810–2.152)	0.265		
D-dimer	3.113 (1.681–5.762)	<0.0001*	1.458 (0.643–3.310)	0.367
FBG	1.054 (0.919–1.208)	0.452		
Triglycerides	0.549 (0.321–0.939)	0.029*	1.156 (0.139–9.598)	0.893
TC	0.852 (0.607–1.194)	0.351		
LDL-c	0.918 (0.584–1.444)	0.712		
HDL-c	0.443 (0.107–1.841)	0.263		
LDL-c-to-HDL-c ratio	1.056 (0.651–1.714)	0.826		
APOA	0.142 (0.025–0.806)	0.028*	0.037 (0.000–25.781)	0.323
APOB	0.880 (0.204–3.787)	0.863		
APOA-to-APOB ratio	0.343 (0.101–1.166)	0.087		
Lipoprotein(a)	1.009 (0.995–1.022)	0.206		
Homocysteine	0.993 (0.962–1.026)	0.675		
Uric acid	1.000 (0.996–1.004)	0.954		
Interleukin-8	1.691 (1.331–2.149)	<0.0001*	1.638 (1.064–2.522)	0.025*
TSG-6	1.157 (1.249–1.975)	<0.0001*	2.171 (1.152–4.093)	0.017*
Dual antiplatelet therapy	0.162 (0.036–0.720)	0.017*	0.264 (0.002–42.701)	0.608
Intravenous thrombolysis	2.290 (0.880–5.959)	0.090		
Endovascular therapy	6.024 (2.111–17.192)	0.001*	0.404 (0.017–9.835)	0.578

*P < 0.05.

OR, odds ratio; CI, confidence interval; BMI, body mass index; SBP, systolic blood pressure; DBP, diastolic blood pressure; NIHSS, National Institute of Health Stroke Scale; ASTRAL, Acute Stroke Registry and Analysis of Lausanne; FBG, fasting blood glucose; TC total-cholesterol; LDL, low-density lipoprotein-cholesterol; HDL, high-density lipoprotein-cholesterol; APOA, apolipoprotein A; APOB, apolipoprotein B; TSG-6, tumor necrosis factor-stimulated gene-6.

With an AUC of 0.795 (95% CI, 0.702–0.887; *P* < 0.0001), TSG-6 concentration showed a significant discriminatory ability to assess non-cardioembolic AIS prognosis (**Figure 4B**), and 10.21 ng/ml (sensitivity, 0.829; 1-specificity, 0.283) was the cutoff value with a higher true-positive rate and a lower false-positive rate for predicting 3-month outcome in non-cardioembolic AIS. Interestingly, TSG-6-to-IL-8 ratios were significantly higher in the favorable outcome group compared with those in the unfavorable functional outcome group [1.94 (IQR, 1.68–2.39] vs. 1.22 (IQR, 0.38–1.54) ng/ml; *P* < 0.0001; **Figure 4C**). The cutoff value of TSG-6-to-IL-8 ratio for predicting the 3-month prognosis of non-cardioembolic AIS (1.505) by ROC curve gained a relatively high true-positive rate (sensitivity, 0.743) and a low false-positive rate (1-specificity, 0.081). The AUC value was 0.873 (95% CI, 0.795–0.951; *P* < 0.0001; **Figure 4D**).

## Discussion

This is the first study to investigate the association between circulating TSG-6 and stroke in humans. In the end, we selected non-cardioembolic stroke patients as the study population, mainly because there were too few patients with cardioembolic stroke that could be included in the study. Given this, cardioembolic, undetermined, and other stroke subtypes were removed. This proportion of subtype was in line with that in Asian populations. The proportion of cardioembolic stroke was about 10% in Chinese patients (2). We found that plasma TSG-6 levels in non-cardioembolic AIS patients significantly increased than those in controls. Likewise, Watanabe et al. (7) showed that plasma TSG-6 levels were significantly elevated in ACS. Moreover, the presence of TSG-6 expression was observed in the abdominal tunica media of AAA patients (8). TSG-6 is generally upregulated wherever there is inflammation to mediate many of immunomodulatory and reparative activities (6). We speculate that TSG-6 may act as a downregulator of the negative effects carried by proinflammatory cytokines to suppress an overreactive inflammation and maintain the dynamic balance of inflammatory mediator release in AIS. Further multiple comparisons between the control, LLA, and SVO groups showed significant increases of TSG-6 levels in both stroke subtypes, and the LAA group had meaningfully higher levels than the SAO group. Inflammation is well-known throughout the process of atherosclerotic plaque formation, destabilization, and rupture (24). TSG-6 contributes to prevent atherosclerosis by suppressing the inflammatory responses and oxidized LDL-induced foam cell formation and the migration and proliferation of vascular smooth muscle cells (25). Additionally, both animal and human studies have shown the expression of TSG-6 in atherosclerotic lesions, for instance, its localization in rat neointima after arterial injury, rabbit carotid atherosclerotic plaques, and human atherosclerotic fibrous cap (7, 8, 26, 27). We speculate that elevated circulating TSG-6 may reflect the activation of ongoing inflammation processes due to ruptured atherosclerotic plaque. As shown in **Figure 1**, the level differences between LLA patients and control subjects were greater than the differences between SVO patients and control subjects. Therefore, we hold the opinion that, for one thing, the overall severity of the condition of LLA patients was higher than that of SVO patients; for another, the atherosclerosis degree of LLA stroke was probably more serious than SVO stroke.

Our present study had suggested that a high expression of TSG-6 was probably associated with non-cardioembolic AIS. To further explore its potential involvement in pathophysiological processes in AIS, we analyzed an AIS dataset from the GEO to extract 782 DEGs that may be AIS-related and *TNFAIP6*-correlated genes. Previous studies have confirmed that TSG-6 was abundantly expressed in human monocyte-derived macrophages (7) and TSG-6 is a secretory protein (6). Here, these samples were derived from peripheral blood mononuclear cells (PBMCs), whereas our samples were peripheral plasma. Therefore, we hypothesize that the expression of TSG-6 in PBMCs might roughly match with their plasma levels in the peripheral plasma, or say, between the two, there might exist a similar trend. Subsequently, pathway and function analysis indicated that the DEGs were associated with response to immunoinflammatory response, environmental information processing, atherosclerosis, production and activity of cytokine including chemokine, leukocyte functional activity, and the like. These biological processes above were in accordance with previous research on TSG-6 (6). Further analysis selected 17 targets deemed as closely interacting with TNFAIP6 in AIS, of which CXCL1, IL-8, and EGF were the top 3 with a high degree. CXCL-1 and IL-8 are both potent neutrophil chemokines playing noteworthy roles in AIS pathophysiology (28, 29). Interestingly, Losy et al. (28) found no increase of serum CXCL1 levels in AIS patients within 24 h after onset, instead advocated CXCL1 local but not systemic production of inflammatory response to AIS. IL-8 is mainly produced by activated monocytes, macrophages, and endothelial cells and acts as a potential polymorphonuclear leukocyte chemotaxis to induce neutrophils to accumulate at ischemic sites, thereby causing inflammation and edema (29, 30). It was known that IL-8 was not merely an independent risk factor affecting AIS prognosis, but its differences could be detected in peripheral blood (31). Several studies have shown that neutrophil recruitment, higher neutrophil-to-lymphocyte ratio, and neutrophil extracellular traps all predict clinical worsening in AIS (32–34). A previous study suggested that TSG-6 inhibited neutrophil migration *via* direct interaction with IL-8 *in vitro* (35). Therefore, we speculate that there might be a connection between TSG-6 and IL-8 in peripheral blood of non-cardioembolic AIS patients. A Spearman correlation analysis demonstrated that TSG-6 levels were positively correlated with NIHSS and ASTRAL score at admission, lesion volume, neutrophil count, neutrophil-to-lymphocyte ratio, and IL-8 level. Among the six positive correlation variables, the IL-8 level had the strongest correlation with the TSG-6 level. Besides, by analyzing the GSE22255 dataset, *CXCL8* expression was upregulated in the *TNFAIP6*-high expression group. These all supported our speculation.

By further comparison, we first raised that elevated TSG-6 levels were associated with 3-month poor prognosis of non-cardioembolic AIS, which was similar to the observation that with increased TSG-6 levels, myocardial infarction and major adverse cardiovascular events (MACE) were more likely to occur, including cardiovascular death, heart failure, acute myocardial infarction, post-infarction angina, and ischemic stroke in patients with acute coronary syndrome during a period of 4 years after onset (25). By multivariable logistic analysis, TSG-6 remained an independent relevant factor for the functional outcome, which suggested that plasma TSG-6 level might be an emerging predictor of short-term prognosis in non-cardioembolic AIS and could help to screen for high-risk patients who need aggressive monitoring and treatment. Nonetheless, these results may contradict the past perceptions of TSG-6, like diverse tissue protective and anti-inflammatory properties. Yet some protective cytokines in AIS such as interleukin-37 (36) and hepatic growth factor (37) exhibited similar results. Inflammatory processes could concomitantly induce both beneficial and detrimental effects after AIS (5). Elevated TSG-6 level hinted that TSG-6 acting as a compensation index to anti-inflammatory action reflected the severity and fighting ability of adverse inflammatory response. By ROC analysis, two cutoff values of plasma TSG-6 concentration for diagnosis and prognosis were raised. Our best predictive value in non-cardioembolic AIS was slightly lower than the previous study of ACS (9.5 ng/ml) (25). In our study, we considered that the TSG-6 reference value of prognosis assessment was more obvious than that of diagnosis. This can be directly seen from the specificity of cutoff values for diagnosis (specificity, 0.550) and prognosis (specificity, 0.717) assessment, whereas the sensitivities of the two cutoff values were similar. Having a quite strong correlation between the levels of TSG-6 and IL-8 in our study and the known antagonistic relationship between the two (35), we further studied the possible changes in the ratio of the two for prognosis. Interesting, TSG-6-to-IL-8 ratio significantly decreased in the 3-month unfavorable outcome group of non-cardioembolic AIS patients than that in the favorable outcome group. Nevertheless, either elevated TSG-6 level or IL-8 level may predict poor prognosis (29), and a higher TSG-6-to-IL-8 ratio might get a better outcome. Besides, the cutoff value of TSG-6-to-IL-8 ratio for predicting the 3-month prognosis of non-cardioembolic AIS gained a relatively high true-positive rate and low false-positive rate. Hence, there is a relatively bold guess that a non-cardioembolic AIS individual with a lower TSG-6 level and a higher IL-8 level might get a poorer outcome due to lack of compensatory protection, which needs further consideration and discussion. These results implied several possible feedback or response mechanisms between the two. Although no consensus was made about how much a marker must add to clinical models in terms of discrimination and reclassification improvements, this study demonstrated some supporting evidence of TSG-6 in non-cardioembolic AIS diagnosis and the value of prognosis assessment and provided a potential target for non-cardioembolic AIS treatment.

Our study has several limitations that should be considered. First, the number of subjects was small and our work lacks long-term clinical outcome data. Second, the study population had geographical limitations, so that the findings should be extrapolated to other populations cautiously. Further studies with larger sample sizes from multiple centers might be more persuasive to validate our findings. Third, plasma TSG-6 concentrations were tested only once at admission, so we were unable to study the variation in plasma TSG-6 levels among non-cardioembolic AIS patients. Fourth, in samples for screening IL-8 as a related factor, the database samples did not highly match with our samples. Although we explained the reason for selecting this method in our previous discussion, the results are more reliable with more consistent sample types and sources. Fifth, due to limitations of environmental, regional, and other factors, we were unable to perform face-to-face follow-up. Differences in cognition, description, and cooperation degree of patients and their relatives increased the bias of outcome assessment. Finally, inflammatory response after AIS is a complicated regulatory network, in which each biomarker potentially influences discrimination, reclassification, and accuracy of prediction. The association between TSG-6 and other inflammatory factors deserves to explore more.

## Conclusions

Our findings first demonstrated that plasma TSG-6 level was a novel indicator for non-cardioembolic AIS diagnosis and 3-month prognosis. The TSG-6 reference value of prognosis assessment was more obvious than that of diagnosis. Plasma TSG-6 and IL-8 levels manifested positive correlations at the early stage of non-cardioembolic AIS. Underlining some potential response mechanisms of TSG-6 and IL-8, elevated TSG-6-to-interleukin-8 ratio might suggest a 3-month favorable outcome. These results provide vital clues for both basic study and clinical application of non-cardioembolic AIS. Further research into the relationship between TSG-6 and ischemic stroke is necessary and warranted.

## Data Availability Statement

The datasets used during the current study are available from the corresponding author on reasonable request.

## Ethics Statement

The studies involving human participants were reviewed and approved by the local Ethics Committee of the First Affiliated Hospital of Harbin Medical University. The patients/participants provided their written informed consent to participate in this study.

## Author Contributions

YQ prepared the study protocol; collected, analyzed, and interpreted the data; and prepared the manuscript. FY and FM collected and analyzed the data. XC, QZ, and TY collected the data. SW interpreted the data. YP prepared the study protocol, analyzed and interpreted the data, and supervised the study. All authors read and approved the final manuscript.

## Funding

This research was supported by grants from the Opening Foundation of State Key Laboratory of Cognitive Neuroscience and Learning, Beijing Normal University (CNLZD1303 and CNLZD1701) and the National Natural Science Foundation of China (No.32030045).

## Conflict of Interest

The authors declare that the research was conducted in the absence of any commercial or financial relationships that could be construed as a potential conflict of interest.

## Publisher’s Note

All claims expressed in this article are solely those of the authors and do not necessarily represent those of their affiliated organizations, or those of the publisher, the editors and the reviewers. Any product that may be evaluated in this article, or claim that may be made by its manufacturer, is not guaranteed or endorsed by the publisher.

## Supplementary Material

Supplementary Figure 1 Standard curve of TSG-6 and IL-8.

Supplementary Table 1 Patient’s characteristics stratified by quartiles of TSG-6 plasma Concentration.

Supplementary Data Sheet 1 Differently expressed genes correlated with *TNFAIP6* in AIS.

Supplementary Table 2 AIS-related targets with closely relevant to TNFAIP6/TSG-6.

## Abbreviations

TSG-6/TNFAIP6, tumor necrosis factor-stimulated gene-6; AIS, acute ischemic stroke; IL-8: interleukin-8; SD, standard deviation; OR, odds ratio; CI, confidence interval; IQR, interquartile range; ROC, receiver-operator characteristic; AUC, area under the curve; LLA, large-artery atherosclerosis; SAO, small-artery occlusion; ELISA, enzyme-linked immunosorbent assay; mRS, modified Rankin Scale; BMI, body mass index; NIHSS, National Institutes of Health Stroke Scale; ASTRAL, Acute Stroke Registry and Analysis of Lausanne; SBP, systolic blood pressure: DBP, diastolic blood pressure; NLR, neutrophil-to-lymphocyte ratio; FBG, fasting blood glucose; LDL, low-density lipoprotein; HDL, high-density lipoprotein; APOA, apolipoprotein A; APOB, apolipoprotein B; Lpa, Lipoprotein(a); HCY, homocysteine; UA, uric acid; DAPT, dual antiplatelet therapy; mTICI, modified thrombolysis in cerebral infarction; rt-PA, recombinant tissue plasminogen activator; u-PA, urokinase plasminogen activator; KEGG, Kyoto Encyclopedia of Genes and Genomes; GO, Gene Ontology; PPI, protein–protein interaction; DEGs, differentially expressed genes.
