# Supplementary figures and images for: Plasma Concentration of Tumor Necrosis Factor-Stimulated Gene-6 as a Novel Diagnostic and 3-Month Prognostic Indicator in Non-Cardioembolic Acute Ischemic Stroke
Source: Front Immunol. 2022 Feb 10;13:713379. doi: 10.3389/fimmu.2022.713379 (PMC8868935; doi:10.3389/fimmu.2022.713379)

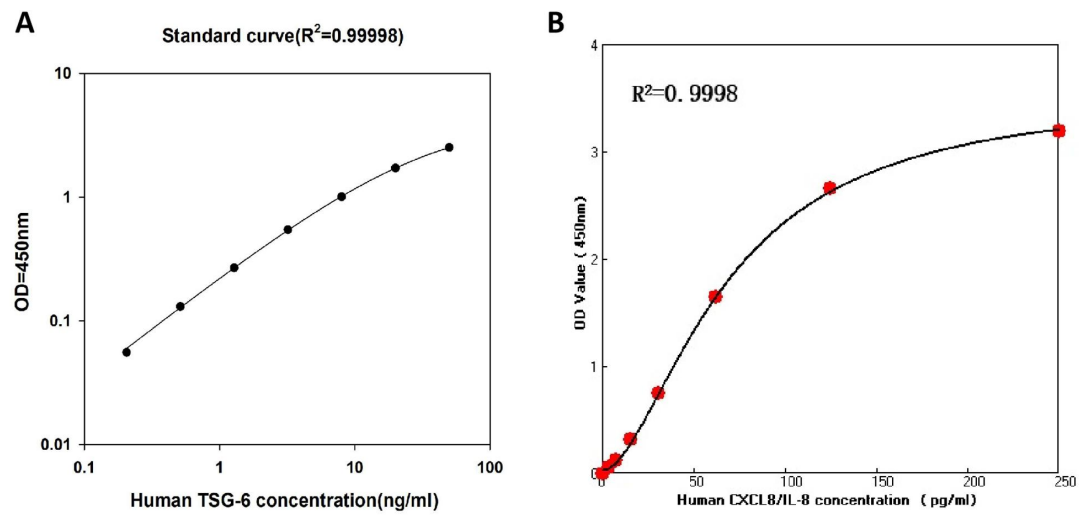

**Supplementary Figure 1 | Standard curve. (A) TSG-6 (B) Interleukin-8.**

Supplement: Supplementary Figure 1 — Standard curve of TSG-6 and IL-8. [file Image_1.pdf]
