# Supplementary material for: Plasma Concentration of Tumor Necrosis Factor-Stimulated Gene-6 as a Novel Diagnostic and 3-Month Prognostic Indicator in Non-Cardioembolic Acute Ischemic Stroke
Source: Front Immunol. 2022 Feb 10;13:713379. doi: 10.3389/fimmu.2022.713379 (PMC8868935; doi:10.3389/fimmu.2022.713379)
Supplement: Supplementary Table 1 — Patient’s characteristics stratified by quartiles of TSG-6 plasma Concentration. [file Table_1.docx]

| **Supplementary Table 2 \|** Patient's characteristics stratified by quartiles of TSG-6 plasma concentration | | | | | | |
| --- | --- | --- | --- | --- | --- | --- |
| **TSG-6 plasma level (ng/ml)** | **Total AIS Patients**  **9.653(8.709-10.727)**  **(n=134)** | **Q1**  **<8.709**  **(n = 33)** | **Q2**  **8.709-9.653**  **(n = 34)** | **Q3**  **>9.653-10.727**  **(n = 34)** | **Q4** >10.727 **(n = 33)** | ***P* value** |
| Sex, Male(%)  Female | 97(72.39)  37 | 19(57.58)  14 | 24(70.59)  10 | 29(85.29)  5 | 25(75.76)  8 | 0.082 |
| Age, years(SD) | 64.14 ± 10.09 | 64.27 ± 8.371 | 62.41 ± 9.372 | 63.5 ± 12.936 | 66.45 ± 8.99 | 0.415 |
| BMI, kg/m^2^(SD) | 25.22 ± 3.35 | 24.67 ± 3.36 | 26.08 ± 3.35 | 25.35 ± 3.39 | 24.73 ± 3.26 | 0.274 |
| **Vascular risk factors(yes), n (%)** |  |  |  |  |  |  |
| Previous stroke/TIA | 75(55.97) | 13(39.39) | 21(61.76) | 21(61.76) | 20(60.61) | 0.188 |
| Hypertension | 86(64.18) | 18(54.55) | 24(70.59) | 19(55.88) | 25(75.76) | 0.181 |
| Diabetes mellitus | 40(29.85) | 11(33.33) | 9(26.47) | 8(23.53) | 12(36.36) | 0.848 |
| Cardiovascular disease | 27(20.15) | 5(15.15) | 9(26.47) | 5(14.71) | 8(24.24) | 0.483 |
| Alcohol drinking | 71(52.99) | 13(39.39) | 19(55.88) | 21(61.76) | 18(54.55) | 0.302 |
| Current smoker | 75(55.97) | 14(42.42) | 18(52.94) | 21(61.76) | 22(66.67) | 0.205 |
| **Clinical findings (IQR/SD)** |  |  |  |  |  |  |
| SBP, mmHg | 156.00(141.00- 172.50) | 152.00(141.50-177.50) | 152.00(140.00-167.75) | 155.00(139.50-169.25) | 158.00(146.00-186.00) | 0.578 |
| DBP, mmHg | 89.50(80.00-100.25) | 90.00(81.50-100.00) | 91.50(79.75-107.00) | 88.50(79.50-100.25) | 87.00(80.00-96.00) | 0.617 |
| NIHSS at admission | 5.00(3.00-12.00) | 3.00(2.00-5.00) | 4.00(2.75-8.00) | 6.00(3.00-11.25) | 12.00(6.00-15.50) | **<0.0001*** |
| ASTRAL at admission | 20.00(17.75-28.00) | 18.00(15.00-20.50) | 19.00(16.00-23.00) | 20.00(17.75-27.25) | 28.00(21.00-33.00) | **<0.0001*** |
| Lesion volume, ml | 2.19(0.48-22.35) | 0.81(0.29-5.63) | 1.58(0.34-13.11) | 3.30(0.67-32.29) | 19.98(1.45-95.85) | **0.003*** |
| **Laboratory findings (IQR/SD)** |  |  |  |  |  |  |
| Neutrophil, 10^9^/L | 6.23( 4.72- 7.68) | 5.23(4.03-6.50) | 6.30(4.29-7.12) | 6.47(4.59-7.66) | 7.81(5.87-10.32) | **<0.0001*** |
| Lymphocyte, 10^9^/L | 1.55 ± 0.58 | 1.60 ± 0.58 | 1.83 ± 0.70 | 1.39 ± 0.48 | 1.36 ± 0.44 | **0.002*** |
| Neutrophil-to-lymphocyte ratio | 3.94( 2.80- 6.04) | 3.49(2.36-4.82) | 3.06(2.45-4.71) | 4.48(2.92-8.24) | 5.15(4.05-9.50) | **<0.0001*** |
| Platelet, 10^9^/L | 214.50(178.00-256.00) | 234.76 ± 69.10 | 215.85 ± 47.16 | 210.09 ± 48.21 | 217.59 ± 65.62 | 0.552 |
| Fibrinogen, g/l | 2.70( 2.15- 3.19) | 2.61 (2.27-3.21) | 2.70(2.13-3.43) | 2.78(2.27-3.12) | 2.74(2.10-3.21) | 0.983 |
| D-Dimer, mg/l | 0.30(0.19- 0.46) | 0.29(0.19-0.41) | 0.28(0.19-0.43) | 0.31(0.19-0.57) | 0.39(0.20-1.09) | 0.235 |
| FBG, mmol/L | 5.75(4.82-7.48) | 5.64(4.94-7.72) | 5.44(4.62-7.14) | 5.49(4.80-6.94) | 6.36(4.92-7.51) | 0.468 |
| Triglycerides, mmol/L | 1.40( 1.03- 2.14) | 1.29(0.96-1.97) | 1.68(1.27-2.61) | 1.57(1.11-2.52) | 1.32(0.90-1.85) | 0.026 |
| TC, mmol/L | 4.71(4.14 -5.45) | 5.08(4.29-5.99) | 4.58(4.13-5.33) | 4.69(4.13 -5.02) | 4.64(3.99-5.35) | 0.238 |
| LDL-c, mmol/L | 3.13 ± 0.86 | 3.27 ± 0.86 | 3.14 ± 0.87 | 3.04 ± 0.83 | 3.08 ± 0.91 | 0.731 |
| HDL-c, mmol/L | 1.12(0.97 -1.32) | 1.24(1.08-1.48) | 0.99 (0.87-1.23) | 1.12(1.03-1.26) | 1.21(1.02-1.33) | **0.015*** |
| LDL-c- to-HDL-c ratio | 2.77 ± 0.80 | 2.67 ± 0.74 | 3.01 ± 0.81 | 2.71 ± 0.91 | 2.68 ± 0.70 | 0.230 |
| APOA, g/l | 1.17(1.06-1.38) | 1.29(1.13 -1.43) | 1.11 (0.98-1.33) | 1.16(1.07-1.37) | 1.19(1.04-1.39) | 0.096 |
| APOB, g/l | 1.02 ± 0.27 | 1.05 ± 0.27 | 1.03 ± 0.28 | 1.00 ± 0.25 | 0.99 ± 0.27 | 0.795 |
| APOA-to-APOB ratio | 1.20( 1.02- 1.44) | 1.20 (1.04-1.54) | 1.21(0.97-1.32) | 1.17(0.94-1.64) | 1.22(1.05-1.44) | 0.835 |
| Lipoprotein(a), mg/dl | 18.28(8.64-42.99) | 11.48(5.34-31.14) | 18.16(11.50-34.81) | 26.16(11.53-45.88) | 17.67(6.78-58.32) | 0.099 |
| Homo-cysteine, umol/L | 14.70(11.98-19.88) | 14.20(11.50-14.82) | 14.75(12.25-20.54) | 16.52(12.90-26.58) | 15.40(12.70-20.75) | **0.044*** |
| Uric acid, umol/L | 339.87 ± 93.79 | 318.20(268.15-385.50) | 363.30(301.75-437.23) | 310.60(276.20-383.43) | 326.50(279.65-369.40) | 0.233 |
| Interleukin-8, pg/ml | 5.34(4.18-7.06) | 4.22(3.61-5.21) | 4.82(3.70-5.61) | 5.57(4.33-7.92) | 8.69(5.99-18.43) | <**0.0001*** |
| **Therapeutic measures, n** |  |  |  |  |  |  |
| Anti-platelet drugs  Mono antiplatelet therapy  Dual antiplatelet therapy | 133  104  29 | NA | NA | NA | NA | - |
| Statins | 132 | NA | NA | NA | NA | - |
| Single intravenous thrombolysis  Rt-PA  U-PA | 18  6  12 | NA | NA | NA | NA | - |
| Single endovascular therapy  Mechanical thrombectomy  Angioplasty  Mechanical thrombectomy with angioplasty  Mechanical thrombectomy with angioplasty and stenting | 14  9  1  2  2 | NA | NA | NA | NA | - |
| Bridging thrombolysis  Rt-PA combined with mechanical thrombectomy  Rt-PA combined with angioplasty  U-PA with mechanical thrombectomy | 4  2  1  1 | NA | NA | NA | NA | - |
| **mTICI score after endovascular therapy, n** |  |  |  |  |  |  |
| mTICI<2b  mTICI2b-3 | 2  16 | NA | NA | NA | NA | - |
| Note: *P < 0.05.  *Abbreviations: BMI, body mass index; IQR, inter quartile range; SBP, systolic blood pressure; DBP, diastolic blood pressure; NIHSS, National Institutes of Health Stroke Scale; ASTRAL,* *Acute Stroke Registry and Analysis of Lausanne; FBG, fasting blood glucose; TC total-cholesterol; LDL-c, low-density lipoprotein-cholesterol; HDL-c, high-density lipoprotein-cholesterol; APOA, apolipoprotein A; APOB, apolipoprotein B; TSG-6, tumor necrosis factor-stimulated gene-6; mTICI, Modified thrombolysis in cerebral infarction; Rt-PA, recombinant tissue plasminogen activator; U-PA, urokinase plasminogen activator.* | | | | | | |
