# Supplementary material for: Plasma Concentration of Tumor Necrosis Factor-Stimulated Gene-6 as a Novel Diagnostic and 3-Month Prognostic Indicator in Non-Cardioembolic Acute Ischemic Stroke
Source: Front Immunol. 2022 Feb 10;13:713379. doi: 10.3389/fimmu.2022.713379 (PMC8868935; doi:10.3389/fimmu.2022.713379)
Supplement: Supplementary Table 2 — AIS-related targets with closely relevant to TNFAIP6/TSG-6. [file Table_2.docx]

| **Supplementary Table 1 \|** Targets at first-stage nodes related to TSG-6/TNFAIP6 in AIS | | | | | | | | | |
| --- | --- | --- | --- | --- | --- | --- | --- | --- | --- |
| node_name | MCC | DMNC | MNC | Degree | EPC | BottleNeck | EcCentricity | Closeness | Radiality |
| CXCL1 | 16128 | 0.63094 | 15 | 15 | 7.835 | 2 | 0.5 | 16 | 2.05882 |
| CXCL8 | 15960 | 0.68975 | 13 | 13 | 7.603 | 1 | 0.5 | 15 | 1.94118 |
| EGF | 12390 | 0.60034 | 13 | 13 | 7.588 | 3 | 0.5 | 15 | 1.94118 |
| IL6 | 15840 | 0.73175 | 12 | 12 | 7.193 | 1 | 0.5 | 14.5 | 1.88235 |
| PPBP | 3168 | 0.66175 | 11 | 11 | 7.013 | 2 | 0.5 | 14 | 1.82353 |
| PTGS2 | 12960 | 0.72963 | 11 | 11 | 7.248 | 1 | 0.5 | 14 | 1.82353 |
| IL1B | 7920 | 0.73825 | 10 | 10 | 7.053 | 1 | 0.5 | 13.5 | 1.76471 |
| PTX3 | 3000 | 0.65844 | 10 | 10 | 7.007 | 1 | 0.5 | 13.5 | 1.76471 |
| LPAR1 | 156 | 0.49567 | 8 | 8 | 5.837 | 2 | 0.5 | 12.5 | 1.64706 |
| THBS1 | 2880 | 0.75809 | 8 | 8 | 6.43 | 1 | 0.5 | 12.5 | 1.64706 |
| AREG | 10080 | 0.78725 | 8 | 8 | 6.192 | 1 | 0.5 | 12.5 | 1.64706 |
| EREG | 5040 | 0.76834 | 7 | 7 | 5.96 | 1 | 0.5 | 12 | 1.58824 |
| IL1RN | 720 | 0.71324 | 6 | 6 | 5.303 | 1 | 0.5 | 11.5 | 1.52941 |
| CDA | 144 | 0.61814 | 6 | 6 | 5.039 | 1 | 0.5 | 11.5 | 1.52941 |
| IDH1 | 144 | 0.61814 | 6 | 6 | 5.328 | 1 | 0.5 | 11.5 | 1.52941 |
| RHOB | 12 | 0.47366 | 4 | 4 | 3.76 | 1 | 0.5 | 10.5 | 1.41176 |
| TM2D3 | 6 | 0.46346 | 3 | 3 | 3.306 | 1 | 0.5 | 10 | 1.35294 |
| Target with higher degree represents more related to TSG-6/TNFAIP6 in AIS. *AIS, acute ischemic stroke; TSG-6, tumor necrosis factor-stimulated gene-6.* | | | | | | | | | |
